# Supplementary material for: Neutralising Antibodies against Enterovirus and Parechovirus in IVIG Reflect General Circulation: A Tool for Sero-Surveillance
Source: Viruses. 2021 May 29;13(6):1028. doi: 10.3390/v13061028 (PMC8228535; doi:10.3390/v13061028)
Supplement: Supplementary file 1 [file viruses-13-01028-s001.zip › viruses-1214386-supplementary.pdf]

Table S1. Selected *Entero*- and *Parechovirus* strain characteristics. EV; enterovirus, PeV; parechovirus, CVA; coxsackievirus A, CVB; coxsackievirus B, E; echovirus

Virus species	Virus genotype	Virus strain	Year of isolation	Isolation specimen	Source
Enterovirus A	EV-A71C1	529491	2015	Faeces	Amsterdam UMC
Enterovirus A	EV-A71C4	75-Yamagata-03	2003	Unknown	Yamagata Prefectural Institute of Public Health
Enterovirus A	CVA6	152776	2011	Vesicle	Amsterdam UMC
Enterovirus A	CVA16	251361	2012	Faeces	Amsterdam UMC
Enterovirus B	E6	252446	2012	Faeces	Amsterdam UMC
Enterovirus B	E9	626196	2016	Faeces	Amsterdam UMC
Enterovirus B	E11	530568	2015	Faeces	Amsterdam UMC
Enterovirus B	E18	733636	2017	Faeces	Amsterdam UMC
Enterovirus B	E25	719302	2017	Faeces	Amsterdam UMC
Enterovirus B	E30	051601	2010	Faeces	Amsterdam UMC
Enterovirus B	CVB5	734864	2017	Faeces	Amsterdam UMC
Enterovirus B	CVA9	150776	2011	Throat	Amsterdam UMC
Enterovirus C	CVA11	715883	2017	Eye fluid	LUMC
Enterovirus C	CVA13	HWE002p105	1988	Unknown	RIVM
Enterovirus C	CVA20	HWE002p105	1988	Unknown	RIVM
Enterovirus C	CVA24	HWE002p105	1988	Unknown	RIVM

Enterovirus D	EV-D68	152313	Unknown	Unknown	RIVM
Parechovirus A	PeV-A1	251386	2012	Faeces	Amsterdam UMC
Parechovirus A	PeV-A3	160929	2010	Faeces	Amsterdam UMC
Parechovirus A	PeV-A4	651432	2016	Faeces	Amsterdam UMC
Positive control	PeV-A1	Harris B	1956	Rectal swab	Amsterdam UMC

Table S2. Neutralising antibody titres against the different *Entero-* and *Parechovirus* strains in the different IVIG batches. GMT; geometric mean titre, IVIG; intravenous immunoglobulins, nAb titre; neutralizing antibody titre, EV; enterovirus, PeV; parechovirus, CVA; coxsackievirus A, CVB; coxsackievirus B, E; echovirus, TCID50; 50% tissue culture infective dose, NA; not applicable

Virus species	Virus genotype	Cell line	TCID 50	Chloroform treatment	IVIG NL 2010	IVIG NL 2017	IVIG Vietnam 2011
	EV-A71C1	RD	268	Yes	54	152	76
	EV-A71C4	RD	100	No	128	256	181
	CVA6	RD	64	No	256	169	32
	CVA16	RD	268	Yes	91	512	512
Enterovirus A	**GMT**				113	241	123
	E6	HT	46	No	181	362	181
	E9	HT	56	No	256	512	362
	E11	HT	32	No	181	362	256
	E18	Hel	46	Yes	181	362	181
	E25	HT	39	No	362	256	304
	E30	RD	105	No	45	97	91
	CVB5	HT	32	No	362	724	724
	CVA9	HT	246	No	1024	861	1024
Enterovirus B	**GMT**				235	373	298
	CVA11	HT	37	Yes	2	2	2
Enterovirus C	CVA13	Hel	98	Yes	2	2	2

	CVA20	Hel	215	Yes	2	2	2
	CVA24	NA	NA	NA	NA	NA	NA
Enterovirus D	EV-D68	RD	127	No	41	128	128
	PeV-A1	HT	372	No	1024	1024	1024
	PeV-A3	LLC	158	Yes	49	152	49
	PeV-A4	HT	158	No	362	256	609
Parechovirus A	**GMT**				263	342	313
Positive control	PeV-A1	HT	100	No	2048	1024	362
